# Supplementary material for: The effects of genital myiasis on the diversity of the vaginal microbiota in female Bactrian camels
Source: BMC Vet Res. 2022 Mar 5;18:87. doi: 10.1186/s12917-022-03189-5 (PMC8897907; doi:10.1186/s12917-022-03189-5)
Supplement: Supplementary file 5 — Additional file 5. [file 12917_2022_3189_MOESM5_ESM.zip › MPL201709200_16s_yy/Treat1/css/js_css/jsPDF-master.zip!/jsPDF-master/examples/test_from_html.html]

Test HTML


<html>
<head>
</head>
<body>
<div>black</div>
<div>black (should not have color parameter in PDF source)</div>
<div style='color: red; font-size: 30px'>hello</div>
<div style='color: rgb(0, 255, 0); font-size: 30px'>hello</div>
<div style='color: #0000FF; font-size: 30px'>hello</div>
<div class='important'>hello</div>
<div>black</div>
<div>black (should not have color parameter in PDF source)</div>
</body>
</html>
